# Consumer Acceptance of a Ready-to-Eat Meal during Storage as Evaluated with a Home-Use Test

**DOI:** 10.3390/foods10071623

**Published:** 2021-07-13

**Authors:** Maria Laura Montero, Dolores Garrido, R. Karina Gallardo, Juming Tang, Carolyn F. Ross

**Affiliations:** 1 School of Food Science, Washington State University, Pullman, WA 99164, USA; maria.montero@wsu.edu; 2 National Center for Food Science and Technology (CITA), University of Costa Rica, San José 11501-2060, Costa Rica; 3 School of Economic Sciences, Washington State University, Pullman, WA 99164, USA; garridom@union.edu (D.G.); karina_gallardo@wsu.edu (R.K.G.); 4 Department of Economics, Union College, Schenectady, NY 12305, USA; 5 School of Economics, Puyallup Research and Extension Center, Washington State University, Puyallup, WA 98371, USA; 6 Department of Biological Systems Engineering, Washington State University, Pullman, WA 99164, USA; jtang@wsu.edu

**Keywords:** home-use test, ecological validity, jambalaya, online auction

## Abstract

A home-use test (HUT) is one method that provides a measure of ecological validity as the product is consumed in home under common daily use circumstances. One product that benefits from being evaluated in-home are ready-to-eat (RTE) meals. This study determined consumer acceptance of microwave-thermally-pasteurized jambalaya, a multi-meat and vegetable dish from American Cajun cuisine, and a control (cooked frozen jambalaya) through an on-line home-use test (HUT) over a 12-week storage period. Paralleling the HUT, an online auction determined consumers’ willingness to pay. The study also explored how the social environment may impact the liking of the meals when a partner of the participants joined the sensory evaluation of the meals. Consumers (*n* = 50) evaluated microwave-processed jambalaya stored at 2 °C and a control (cooked frozen jambalaya stored at −31 °C) after 2, 8 and 12 weeks of storage. Consumer liking of different sensory attributes was measured. Participants could choose to share the meals with a partner as a way to enhance ecological validity. The responses from 21 partners to the sensory-related questions were collected. After the sensory evaluation, the participants bid on the meal they had just sampled. Results showed that processing method (microwave vs. control) did not significantly influence the measured sensory attributes. Only flavor liking decreased over storage time (*p* < 0.05). The inclusion of partners significantly increased (*p* = 0.04) the liking of the appearance of the meals. The mean values of the bids for the meals ranged from $3.33–3.74, matching prices of commercially available jambalaya meals. This study found suggests that the shelf- life of microwave-processed meals could be extended up to 12 weeks without changing its overall liking. The study also shows the importance of exploring HUT methodology for the evaluation of consumers’ acceptance of microwave-processed jambalaya and how including a partner could contribute to enhance ecological validity.

## 1. Introduction

Sensory evaluations are commonly made in confined spaces in sensory laboratories. However, people do not habitually consume their meals in sensory booths, individually partitioned in a room full of strangers, focused on a ballot to assess their food. Rather, they eat at home, in a restaurant or café; sometimes alone, but frequently with family, friends and colleagues [1].

Koster (2003) [2] has shown that testing in a sensory lab regular setup removes the natural consumption setting in which a food is consumed, and thus makes it difficult to elicit accurate data from consumers. Ecological validity is the degree to which a test predicts behaviors in real-world settings or the extent to which the context of the evaluation matches the user’s real context [3]. Test designs that parallel real-life situations produce findings that can be generalized to real life outcomes [4]. To enhance ecological validity, the site of the research can be shifted from the standard sensory booth to a setting closer to a more authentic environment, one that closely approximates the condition in which consumers actually purchase or consume the food products being tested [1,4,5].

Because it remains challenging to evaluate food products in a controlled manner within the consumer’s natural habitat, in recent years researchers have begun to explore novel solutions to provide more authentic environments [1]. The home-use-test (HUT) is one such methodology. In a HUT, consumers prepare, consume, and evaluate samples in their homes, usually for a period of several days [6,7]. HUTs offer tremendous advantages in terms of validity of the data generated, this type of test is less controlled and allows for the evaluation of product attributes under conditions that relate more closely to real-life usage, thereby increasing the validity of data obtained [6,8]. The opinions of other family members or partners can also enter the picture, as they do in everyday use of purchased products.

Various factors, such as social interaction, physical environment and the serving of the product may influence the liking of different food products [9]. Social interaction may play a key role in consumer behavior [7]. Social interaction may explain the reason why different hedonic results could be observed between standardized situation tests (SST), like laboratory test or central location tests (CLT) and HUTs. In SST, the consumption of the product been tested is always individual whereas in HUT it can be social [7]. Clendenen, Herman, and Polivy, 1994 [10] reported that subjects eat more when eating occurred in groups of several people than when they eat alone especially when meal companions are relatives or friends.

The different locations, even when consumers evaluate food under natural conditions, can also impact the acceptance of a food product depending on the circumstances [7]. Accordingly, de Graaf et al. (2005) [11] reported that the predictive ability of laboratory ratings depends on the type of food been evaluated. Lab ratings are more relevant for snacks than for served dishes. A key factor in the explanation of differences in hedonic results between SST and HUT is the way the product is usually eaten [7]. For products that are strongly related to specific contexts and serving size, a HUT might be more useful to determine consumers’ acceptance [6]. Multiple studies have compared CLT versus HUTs in the evaluation of consumers’ acceptance of products such as ready-to-mix protein beverages [6]; cod products [12]; ready-to-heat meals [13]; as well as salted cheese crackers and sparkling water [7].

Auctions offer another ecologically valid method of assessing consumer acceptance of food. Although individual dietary choices are primarily influenced by such considerations as taste, convenience and nutritional value of foods [14] cost has to be considered as a factor in the development of the product. In new product development, it is important to evaluate consumers’ willingness to pay for the product. In laboratory settings or field experiments, such as HUTs, auctions have been intensively employed to elicit willingness to pay. In auctions, products, services or rights are bought and sold through a formal bidding process [15]. An auction is useful to gain knowledge of consumers’ evaluations of a product or brand; thus, auctions can be used to reveal consumers’ valuations to facilitate future pricing decisions [16].

One product that is appropriate for assessment by a HUT and an auction is a RTE meal. Ready-to-eat (RTE) meals are products that are pre-cooked, packaged, and ready for consumption without additional preparation and cooking beyond simple heating [17]. Consumption of RTE meals in the United States has been influenced by the fact that over the past four decades, demand has grown for foods that save households time in meal preparation and cleanup (i.e., “convenience foods”). This type of meal fits very well with the needs of consumers who are looking for convenience in food products [18]. However, there is also a need to develop more nutritious, safe, autochthonous, and quality-enhanced RTE meals.

Within this context, microwave pasteurization offers opportunities for the food industry to produce high quality, safe, frozen and chilled RTE meals [19]. The main advantage of a microwave-assisted pasteurization system (MAPS) over traditional thermal processing systems is reduced processing time; the generation of volumetric heat in this system makes it possible to increase the heat transfer rate and reduce the total heating time by three to five times [20,21]. Thus, MAPS is particularly suitable for pasteurization of pre-packaged, heat-sensitive, multi-component meals that are highly viscous, semisolid, or solid [19]. Montero, Sablani, Tang and Ross (2020) [22] investigated the potential of MAPS to extend the shelf life of RTE fried rice. The authors found that MAPS processing was able to extend the shelf life of a chilled fried rice meal up to 6 weeks when stored at 7 °C, demonstrating the potential of this technology for the RTE industry. Barnett, Sablani, Tang and Ross (2019) [23] evaluated the shelf life of sterilized microwave-processed chicken meals and consumer liking of the meals and found that the overall liking did not vary due to the effect of storage time.

The use of a sensory methodology such as a HUT is highly suitable to evaluate consumers’ liking of RTE meals. Since there is an extra step before the consumption of the meal (e.g., heating via microwave), and it is tested at home this adds a more realistic context to the sensory experience and expectations of consumers because it resembles the way these meals are usually eaten.

Therefore, this study determined consumer acceptance of MAPS-processed jambalaya and a control (cooked and frozen jambalaya) through an on-line HUT over a 12-week storage period. Jambalaya was chosen because it is a multicomponent ready-to-eat meal with three different types of protein ingredients (sausage, chicken and shrimp) and a vegetable-based sauce, making it a complete meal and suitable for microwave processing. Jambalaya, a regional dish of the American South, is a type of RTE meal that is increasingly available nationwide to consumers who are interested in exploring regional and global cuisines [24,25]. Paralleling the HUT, an online auction determined consumers’ willingness to pay. Another goal of the study was to determine the degree to which a manipulation in the social environment of the HUT impacted the level of perceived acceptability on the part of the participants

The study had two hypotheses: (1) the acceptance/liking of different sensory characteristics of MAPS-processed RTE jambalaya would not change significantly during storage as compared to a control (cooked and frozen jambalaya) over a 12-week storage period; (2) ecologically valid measures of consumer acceptance (a modified HUT and an auction) would impact the degree of acceptance of the RTE meals.

## 2. Materials and Methods

### 2.1. Preparatory Steps

#### 2.1.1. Jambalaya RTE Meals Preparation

The formulation and ingredients shown in Table 1 were used in the production of the jambalaya:

The jambalaya was manufactured in a sanitary food preparation room at the School of Food Science facilities (Pullman, WA, USA) according to the formulation presented in Table 1. Over a period of three days, 120 trays were assembled and sealed per day for a total of 360 jambalaya trays. The total time for a batch of 120 trays to be cooked, assembled, stored, and MAPS-processed was two days. The workload was staggered in batches of 120 trays over a four-day period as shown in Table 2.

All the ingredients were weighed and prepared the day before the preparation of the jambalaya. The daily cooking steps for the chicken, sausage, shrimp and sauce are described here.

Chicken: 15 mL of olive oil was added to a deep sauté pan over medium-high heat (level 6). The chicken was added and cooked for 5 min on one side until golden brown. Each piece of chicken was then turned over and cooked for another 5 min until fully cooked (to an internal temperature of 74 °C). The cooked chicken was transferred to a bowl and set aside for 5 min. Then the meat was manually pulled into pieces of approximately 2.54 cm (1 inch). The pulled chicken pieces were transferred to a disposable aluminum foil pan with a lid and then stored at 4 °C until the trays were assembled.

Sausage: These were unpacked, and the edges were cut and discarded. They were sliced into 0.6 cm rounds. Approximately 14–16 slices were obtained from each sausage. A total of 15 mL of olive oil was heated in a pan over medium-high heat (level 6/10). Then the sausage slices were added. They were seared for 2 min on one side. The pan was then removed from the burner and the sausages were turned to the uncooked side. The uncooked side was seared for 30 s. Each batch of cooked sausages was then transferred into a disposable aluminum pan with a lid and stored at 4 °C until the trays were assembled.

Shrimp: The shrimp were thawed by placing 48–54 shrimp on trays the day before the jambalaya preparation, so they could defrost for at least 16 h under refrigerated conditions (4 ± 1 °C). After being thawed, the shells were removed but not the tails. A total of 15 mL of olive oil was heated in a pan over low-medium heat (level 4/10). Then the shrimp were added. The shrimp were seared on one side for 1 min. The pan was then removed from the burner and the shrimp were turned to the uncooked side. Then the shrimp were seared for 30 s on the uncooked side. Each batch of cooked shrimp was then transferred into a disposable aluminum pan with a lid and stored at 4 °C until the trays were assembled.

Sauce: 15 mL of olive oil was added to a large pot over medium-high heat (level 6/10). Then the pre-chopped onion, celery, and *pasilla* pepper were added. The ingredients were cooked until they caramelized (approximately 6 min). Next the chicken broth, Old Bay and Cajun seasoning, tomatoes and Worcestershire sauce were added. The heat was increased and brought to a boil (approximately 15 min). Then the heat was reduced to simmer (low heat level 2–3) for 5–6 min. Each batch of cooked sauce was then transferred to a disposable aluminum pan with a lid and stored at 4 °C until the trays were assembled.

Meal Assembly: After all the meat ingredients (chicken, sausages, and shrimp) and the sauce were prepared, 250 ± g EVOH trays (Silgan PFC, dimensions: 15.5 × 11 × 3 cm) were assembled as described here. The assembled product in each tray consisted of 30 ± 0.5 g of sausages (6 units); 40 ± 0.5 g of shrimp (6 units); 40 ± 0.5 g of pulled chicken; and 140 ± 0.5 g of sauce. Once the trays were assembled, they were sealed with film lids with the same composition reported by Barnett et al. (2019) [23], under the following conditions: 200 °C for 4 s under a 65 mbar vacuum with a 400 mbar nitrogen flush. The sealed trays were then stored at 4 °C.

#### 2.1.2. MAPS and Frozen Meal Processing

MAPS Processing/Freezing: On the day following production, 60 trays of the daily production of 120 were processed through MAPS and the other half were frozen (−35 °C) and used as a control. In total, 180 were frozen as controls and 180 trays were pasteurized in a pilot-scale MAPS in the Food Processing Pilot Plant at Washington State University (WSU), Pullman, WA. A detailed description of MAPS can be found in Tang et al. (2018) [19]. The specific processing conditions used to produce jambalaya in the MAPS are described in the methods section of Montero et al., (2020) [22]. At the time the study was conducted, the MAPS could process 16 trays in one run; thus, there were 12 total runs. After being MAPS-processed, the 180 trays were stored at 2.0 ± 0.5 °C. A Temperature Data Logger RC-5+ (Elitech, CA, USA) was used to track the storage temperature during the whole study.

A total of 180 trays were used as control samples (frozen and stored at −31 °C). The control samples were sealed under conditions identical to those of the MAPS-samples. During the freezing step, the sample trays were placed on boards across the top shelves in a freezer 1 m in front of the evaporator with an air velocity of 1.6 m/s and stored at −31 °C. The storage conditions for the control samples were selected to ensure minimal product changes over the length of the study.

Trays of each type (MAPS and control) were randomly selected and analyzed for microbial, sensory, and chemical properties at 2, 8, and 12 weeks of storage.

Microbial/Safety Testing: At weeks 2, 8 and 12 microbial analyses were performed. MAPS-processed jambalaya and control trays were randomly selected and sent to Micro-chem Laboratories (Seattle, WA, USA). The jambalaya samples were screened for the following pathogens as a way to assure their safety before human consumption, *Bacillus cereus* (Local Instruction); *Salmonella*; *Listeria monocytogenes*; and *E*. *coli* O157:H7 (AOAC 050501). For the analyses of pathogens, a 25 g sample was tested. The following analyses from AOAC International Official Methods of Analysis were used to detect signs of spoilage: aerobic plate count; yeasts and molds; and total coliforms. The results from the microbial testing are presented in Table 3.

Based on the microbial testing results the jambalaya meals were safe for consumption at each of the evaluated time points.

The jambalaya meals were evaluated in two separate sensory evaluations by two different groups of participants, a home-use test and a semi-trained panel evaluation.

#### 2.1.3. Participant Recruitment and Orientation

The study protocol described here received the approval of the WSU Institutional Review Board for conducting tests with human subjects, under the title Consumer Preferences of Jambalaya IRB #16994.

Participant Recruitment and Selection: 50 participants with previous experience in sensory evaluation (18 male, 32 female, ages 21 to 78 years, mean age = 40 years) were recruited through the WSU Sensory Evaluation Listserv. Most of the participants were students, staff, or retirees of WSU and community members living in the Pullman (WA) and Moscow (ID) region.

The participants were recruited based on the following three criteria: expressed liking for and frequency of consumption of RTEs (at least twice a month); not presenting allergies to the jambalaya ingredients; and being available and committed to doing the sensory testing at the three defined time points.

**Figure 1:**
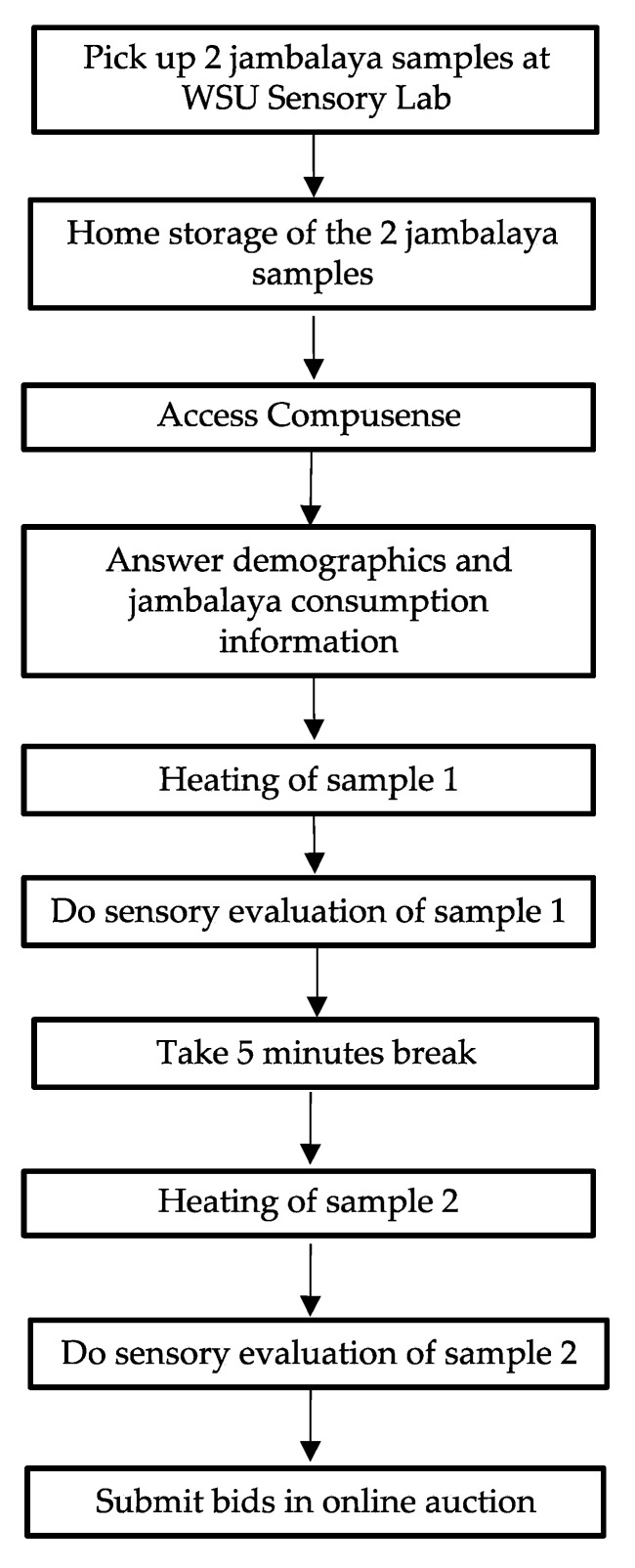
Instructions provided to the panelists in the home-use test evaluation of jambalaya.

Orientation and Procedures for the HUT: The jambalaya samples were tested in a home-use test. A 30 min orientation session was conducted on the same day of the first sensory evaluation time point. The objectives of the session were to explain to the participants the general aim of the study; to provide instructions on how they should manage the two jambalaya samples prior to and during consumption (e.g., heating instructions); to explain how the sensory evaluation was conducted online; and to explain how to participate in the online auction (Figure 1).

Because the jambalaya samples provided a full serving so that two adults could portion out and evaluate the same serving, participants who were able to have one other person evaluate the samples with them (i.e., partner, husband, wife, friend, roommate) were encouraged to do so. The requirements for a partner to participate were to be over 18 years old and not present allergies to the jambalaya ingredients. All participants’ partners signed a consent form in accordance with IRB #16994. A total of 21 partners joined the study (11 male, 10 female, ages 26 to 85 mean age = 44 years). Their answers to the sensory evaluation were collected with a paper-based questionnaire. Partners did not participate in the on-line auction.

As shown in Figure 1, the participants picked up the two jambalaya samples from the Food Science and Human Nutrition Building on the specified evaluation day. At each time point, the samples were provided to the participants in a small cooler that contained the two jambalaya samples packed inside a plastic bag with a sticker that indicated the heating instructions and the order in which the samples should be tested. Each jambalaya meal was assigned a three-digit code so the participants could easily identify each meal. The serving order was randomized across participants. In the heating instructions, the participants were asked to first puncture the tray’s lid on each corner using a knife; then to microwave the meal on Power 9 for 3 min; let the sample rest for 1 min inside the microwave; afterwards to take the tray out of the microwave and to carefully stir the content with a spoon; finally, to transfer the content to a white container so they could easily conduct the sensory evaluation. The participants were indicated to evaluate the jambalaya meals during dinner time, between 5:00 and 9:00 p.m.

The control trays had been thawed in water at room temperature for 8 h before being distributed to the participants, so both the MAPS sample and the control looked the same. The cooler also contained an ice pack (Freez Pak™ Mini, Lifoam, MD, USA) to keep the jambalaya samples at a cool temperature and 2 units of unsalted crackers (Nabisco, NJ, USA) were provided to serve as palate cleansers.

### 2.2. Evaluation Procedures

#### 2.2.1. HUT Evaluation

HUT Scales: Participants in the home-use test used a total of four different scales to evaluate the entrees described here. Question design and data acquisition were accomplished with Compusense^®^ Cloud (Guelph, ON, Canada) software.

A 7-point hedonic scale [13] was used to test the liking/acceptance of different sensory modalities: the overall liking; aroma; overall flavor; texture acceptance of the shrimp, chicken and sausage; and the overall liking.

A 5-point just-about-right (JAR) scale was used to test the spiciness and texture perception of each of the three meat components (shrimp, chicken and sausage).

A 3-point JAR scale about perception of the size of the jambalaya meal was asked at the end of the study. The scale ranged from 1 (=less than I would like) to 3 (=more than I would like). The participants were asked about their perception of the unit/tray size (250 g = 1 serving); the quantity of sauce; the quantity of vegetables; the size of the vegetables; the quantity of each of the meats, shrimp, chicken, and sausage; the level of saltiness; and their preference for tails off the shrimp.

Participants were also asked (open question) to describe the experience participating in the HUT. Comments were collected, revised and categorized into seven groups. The categories were validated by the agreement between two researchers of the study. The categories are the following: enjoyed experience with partner, HUT vs. in-lab evaluation, time flexibility, fun/positive experience, liking of the meals and willingness to pay for the meals.

Willingness to Pay Evaluation: In collaboration with the School of Economics, a complementary study, an online auction was conducted to measure product satisfaction by the willingness of participants to pay for the jambalaya samples. At each of the three evaluation time points (Weeks 2, 8 and 12), after the sensory evaluation component, the participants were asked to submit their bids (i.e., their willingness to pay) for a unit (equivalent to 9 oz-250 g) of each of the jambalaya sample tested. Compensation for doing the sensory evaluations as well as the online auction at each of the three evaluation points totaled $90.00 in cash mailed to the participants. Partners were not included for this component of the study.

The online auction followed a second price auction protocol. The protocol and the benefits of using this type of action to determine the willingness to pay are described by Lusk and Shogren (2007) [26].

The protocol followed in the present study is reported by Garrido et al. (2021) [24]. To determine the winner of the auction, the first step was to randomly select one of the jambalaya samples (control or MAPS). The winner of the auction was the participant who placed the highest bid for the selected sample. The winner received one meal unit of this meal, and in exchange, they had to pay the market price, or the second highest bid. This process was repeated at each of evaluation time points and was done after the sensory testing of the meals.

At the first evaluation time point (2 weeks of storage), no information about the two samples of jambalaya was provided before participants submitted their bids in the auction. The only information provided was the three-digit code or identification number for each meal. At the second and third evaluation time points, two pieces of information were disclosed to the participants before bidding. The order for receiving these two pieces of information was randomized among the participants. At the second time point (8 weeks of storage), the information about the name of the technology used to preserve each jambalaya sample (MAPS versus freezing) was provided to 25 participants. The information about the environmental impacts of the MAPS sample versus the frozen sample was provided to the remaining 25 participants. At the third time point (12 weeks of storage), the information disclosure was reversed. To avoid interfering with the participants’ ratings of the sensory attributes of the meals, the information about the name of the technology and the environmental impacts was disclosed after the sensory testing [24].

#### 2.2.2. Semi-Trained Panel Evaluation

Participant Selection and Orientation: A semi-trained panel (*n* = 10; 8 females, 2 males, ages 23–46) also evaluated the sensory profile of the MAPS-jambalaya and the control with rate-all-that-apply (RATA) questions. All the members of the semi-trained panel had previous experience in conducting sensory evaluation and had participated in multiple descriptive panels conducted at the WSU Sensory Science Center [22].

These evaluations were also done at Weeks 2, 8, and 12 of storage. RATA methodology has been reported to be a valid and reliable sensory profiling tool suitable for semi-trained panels [22,27]. For each session, the control trays were thawed in water at room temperature for 1.5 h. Next, each jambalaya tray (250 g) was warmed at 45–50 °C for 30 min (15 min on each side, top and bottom) with a food warmer (Glo-Ray HATCO Corporation, Milwaukee, WI, USA). Then the trays were opened, and the jambalaya was carefully mixed. A total of 17–20 g of warmed jambalaya was then portioned into plastic cups; each sample was checked to ensure it contained all of the proportionally identical components of the jambalaya (sausage, shrimp, chicken). All samples were evaluated at 40 ± 1 °C. A 30 s break was given after the evaluation of each sample. Filtered water and unsalted crackers (Nabisco, NJ, USA) were provided as palate cleansers. Evaluations were conducted individually, in a discussion room, under white lighting.

**Figure 2:**
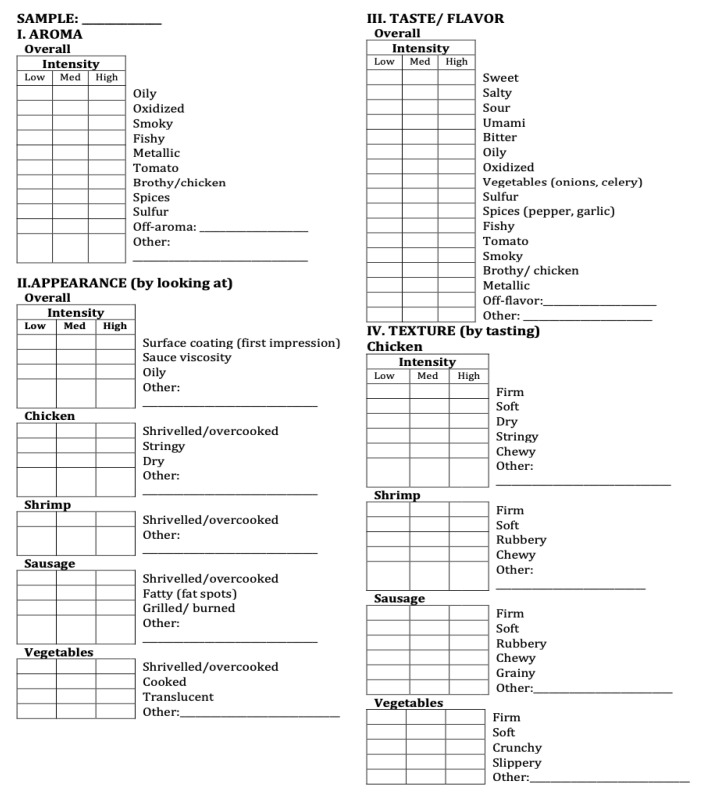

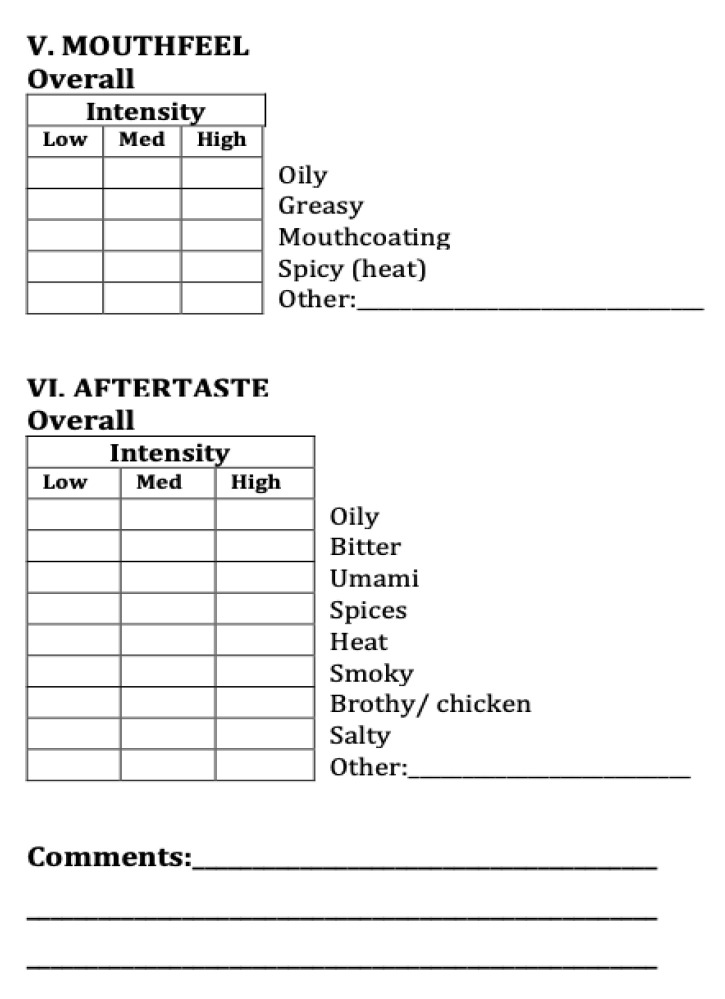
List of the sensory attributes tested for the MAPS-jambalaya and the control with RATA questions.

RATA questions for the jambalaya were divided into six sections: aroma, appearance, taste/flavor, texture, mouthfeel, and aftertaste. As assessors evaluated six sensory modalities-aroma, followed by appearance, taste/flavor, texture, mouthfeel and aftertaste, they checked the terms they considered appropriate to describe the jambalaya samples (Figure 2). The list consisted of 4 to 17 terms, depending on the sensory modality. The terms used for each of the sensory modalities were defined based on pilot work. Assessors then rated the intensity of the selected terms, using a three-point structured scale (low, medium, and high). Answers were collected with a paper-based ballot. The jambalaya samples were coded with three-digit codes and presented in monadic sequential, randomized, balanced order.

### 2.3. Analyses

#### 2.3.1. Sensory Data Analysis

HUT Data: During the 12-week storage period, a repeated measures ANOVA with mixed models was conducted to evaluate the liking results of the different sensory modalities of the jambalaya samples. Processing method, storage time, having a partner and the interaction between processing method*time were analyzed as the fixed factors, with storage time as the repeated factor, and panelists as the subject factor. Means were separated with Tukey’s HSD test. The JAR scale results were interpreted with penalty analysis for each of the meals at the three-evaluation time points (2, 8 and 12 weeks). XLSTAT 2017 (Addinsoft, Paris, France) statistical software was used for all sensory data analyses.

RATA Data: RATA results were analyzed by treating the RATA scores as continuous data and expanding the scale to four points (0, 1, 2, 3 for absent, low, medium, and high, respectively) [22,28,29]. A repeated measures ANOVA with mixed models was conducted. Processing method and storage time and the interaction (treatment*time) were analyzed as the fixed factors, storage time as the repeated factor, and participants as the subject factor. Means were separated with Tukey’s HSD test. Significance was defined as *p* < 0.05.

If the terms were used at a frequency of 20% or less by the assessors, those terms were not considered for analysis [22]. Out of the 82 terms (Figure 2) that comprised the complete list of attributes, 14 were not considered for analysis. The results from the assessors were validated by internal agreement on the rating of the intensity of one of the attributes, as described by Montero et al. (2020) [22].

#### 2.3.2. Online Auction Data Analysis

Data from the online auction were analyzed by a repeated measures ANOVA with mixed models. Treatment and storage time and the interaction (treatment × time) were analyzed as the fixed factors, storage time as the repeated factor, and participants as the subject factor. Means were separated with Tukey’s HSD test (HSD).

## 3. Results and Discussion

The study evaluated two main hypotheses.

**Hypothesis** **1.** *The acceptance/liking of different sensory characteristics of MAPS-processed jambalaya would not change significantly during storage at 2 °C as compared to a control (cooked and frozen jambalaya) over a 12-week storage period.*

**Hypothesis** **2.** *E* *cologically valid measures of consumer acceptance (a modified HUT and an online auction) would impact the degree of acceptance of the RTE meals. Hypothesis 2 employed an exploratory approach regarding how the social environment may impact the liking of RTE jambalaya meals when a partner joins the evaluation of the meals.*

### 3.1. HUT Evaluation

#### 3.1.1. Comparison of Results *n* = 50 vs. *n* = 71: Inclusion of Partners

As a way to enhance ecological validity of the study and test Hypothesis 2, the responses from 21 partners were collected, included and analyzed. The obtained results were compared to the responses of the 50 participants.

In comparing the consumer liking scores of the 50 participants with those of the 71 participants (50 participants + 21 partners), there were no significant differences in the liking scores for most of the tested sensory modalities (Table 4 and Table 5). These results indicate that it is reasonable to include the responses collected from the 21 partners. To have a larger number of responses in a HUT increases the robustness and power of the observed results [30]. It was observed that in certain sensory modalities such as appearance (Table 5) the *p*-value decreased and moved closer to being significant. This result could indicate that it was possible to identify potential difference due to the storage time effect as the number of responses increased.

Two main effects were evaluated with the 71 collected responses. To test Hypothesis 1, the effect of the processing method (MAPS-processed and in chilled storage vs. control, cooked and frozen storage), the effect of the storage time, and their respective interaction on the consumer liking scores for the different sensory modalities were evaluated. The interaction was not significant for any of the sensory modalities; thus, the simple effects were analyzed.

The processing method (MAPS-processed vs. control) did not significantly influence the liking scores that the participants (*n* = 71) assigned to the different sensory attributes that were evaluated (Table 4). These results indicate that the acceptance of multiple sensory attributes was comparable between the MAPS-processed meals and the cooked and frozen (control) meals.

As shown in Table 5, when the storage time effect was evaluated, only flavor liking scores decreased significantly over time (*p* = 0.001, *n* = 71). Considering the meals were evaluated on a 7-point hedonic scale, the liking score for flavor ranged between like slightly and like moderately. For a multicomponent new RTE meal that rating level can be considered as an acceptable/good liking score. On a nine-point hedonic scale, a mean liking score of 7 (like moderately) is usually indicative of highly acceptable sensory quality [31].

The results obtained from the evaluation of the processing method and the storage time indicate the potential of MAPS processing to extend the shelf life of a complex RTE meal such as the jambalaya when stored at 2 °C. Given the increased consumption of RTE nowadays, the food industry is constantly looking for alternative processing techniques that allow for the extension of the shelf of RTE meals and do not require a freezing step. Freezing has been reported as an effective method to extend the shelf life of multiple food products including RTE meals; however, it is energy intensive, and it can affect the texture-related characteristics when freeze-thawing occurs [32]. For this reason, the potential of MAPS-processing seems promising in the processing and conservation of RTE stored under refrigeration conditions.

A paucity of HUT studies have focused on the evaluation of RTE meals consumed in a home setting. A similar HUT study by Olsen et al., (2012) [13] determined that overall liking of the meal drives consumers’ likelihood of buying healthy convenience meals. As in the present study, complex foods were evaluated. Two ready-to-heat meals with multiple components or different ingredients were assessed: (1) salmon fillets with raw vegetables (cauliflower, carrots, and green beans), precooked pasta and pasteurized cream and mushroom sauce; and (2) chicken breast fillets, with raw vegetables (cauliflower, carrots, and green beans), precooked white rice and pasteurized red bell pepper sauce. However, Olsen’s results focused on drivers of overall liking including appearance, odor, amount of ingredients, and flavor. While these attributes were also considerations in our study, our study also focused on the effects of the processing method, storage time and the enhanced social environment in which the food was consumed along with the willingness to purchase of the product.

#### 3.1.2. Willingness to Pay as Evaluated with an Online Auction

To evaluate the participants willingness to pay for the jambalaya meals an online auction was conducted. The auction protocol established that participants received money for placing the bids at each evaluation time point, and because the economic resources were limited, only the 50 participants joined the online auction. The bidding was performed based on the sensory evaluation of each of the jambalaya meals. Information of the processing system was not included at the time of placing the bid at the first time point; this type of information was given at the second and third time points. However, during these two points, the information was provided after the participants conducted the sensory evaluation and prior to submitting their bids [24]. As previously mentioned, the effect of the processing method, storage time and their interaction on the bid values were also analyzed. The interaction was not significant and for this reason the main effects were interpreted. The bid values did not significantly differ (*p* = 0.48) between the MAPS-processed jambalaya and the control, and they were not influenced by the storage time (Table 4).

The bids values assigned by the participants to the meals were comparable to commercially available jambalaya meals. The mean bid values ranged from $3.48–3.74 for the MAPS-processed jambalaya and from $3.33–3.56 for the control.

#### 3.1.3. Effect of Eating with Having a Partner on the Consumer Liking Scores of the Jambalaya Meals

To test Hypothesis 2, the effect of eating with a partner in the liking scores of the sensory characteristics of the meals were also evaluated (Table 6).

It was determined that those participants having a partner gave a significantly higher score (*p* = 0.04) to the appearance of the jambalaya meals. The value was 5.88 vs. 5.54 for those participants without a partner. This liking value is associated with a rating between like slightly and like moderately on a seven-point hedonic scale. Overall, there was a trend in the liking scores of the evaluated sensory attributes; those participants with a partner gave higher scores to the liking of all the evaluated sensory attributes of the meals. Laureati and Pagliarini (2019) [5] defined three main contextual factors that influence eating behavior when conducting consumer testing, the meal (i.e., sensory characteristics); the physical environment (i.e., appropriate location and setting); and the social environment or social interaction (i.e., people present at the experiment). In this study, each of those factors was explored and the social environment seemed to be positively impacted by the partners addition. Piliner, Bell, Kinchla and Hirsch (2003) [33] stated that social interaction has a positive effect on food consumption of naturally created groups but not artificially created. In our study, the partners (spouse, friend, roommate) could be categorized as members of a naturally created group for the participants or that will evoke a more realistic consumption situation [34,35].

Petit and Siefferman (2007) [36] maintain that conducting food testing in naturalistic conditions is more advantageous than in-lab tests due to the realism of the evaluation, but situational tests such as HUTs can be more expensive and time-consuming than in-lab ones. As shown in our study, the addition of a partner could mitigate some of these downsides, mainly the one related to costs. Currently, with COVID-19 restrictions, including responses from partners could represent a simple option for sensory scientists and food companies to increase the number of respondents and enhance ecological validity of the study.

In this study the modified HUT and an online auction seemed to positively impact the degree of acceptance of the RTE meals.

To address Hypothesis 2, on the last evaluation time point the 50 participants were asked to provide feedback about their experience participating in the HUT. The comments were carefully reviewed and divided into seven categories (Table 7). Almost 30% of the participants of the study indicated that they had a positive experience when sharing the evaluation of the meals with their partners. One of the participants mentioned “sharing the samples with my partner is enjoyable, because after we record our ratings individually, we compare and discuss the two samples”.

The category HUT vs. in-lab evaluation shows how over 40% of the participants preferred doing the sensory evaluation of the jambalaya meals at home vs. at the SST or in-lab set up. One of the participants mentioned “I like bringing the samples home to taste because it is more realistic and relevant to how I would actually eat the food”; this comment points to the value of conducting the sensory evaluation under a setup that is more realistic and familiar to the participant. These aspects contribute to the enhancement of the ecological validity of the study [1,36]. Time flexibility was another topic mentioned by the participants in their comments. Almost 15% of their comments indicated that the participants liked being able to take their time and enjoy each sample without feeling rushed. This is another positive component of the in-home evaluation setup. Another category of frequently mentioned comments (42%) was that the participants had a fun or positive experience while participating in the study.

The two final categories were liking of the meal and willingness to pay-related comments. Almost 20% of the participants mentioned liking the meals and almost 10% expressed their willingness to pay for the jambalaya meals if they were available in the market.

Based on the type of comments mentioned by the participants of the HUT, it seems that overall, the HUT was a pleasant, positive experience that allowed them to manage the time for evaluating the meals at their own convenience. This seems like a promising way to accomplish Hypothesis 2.

#### 3.1.4. Potential Improvements on the Jambalaya RTEs Meals

**Figure 3:**
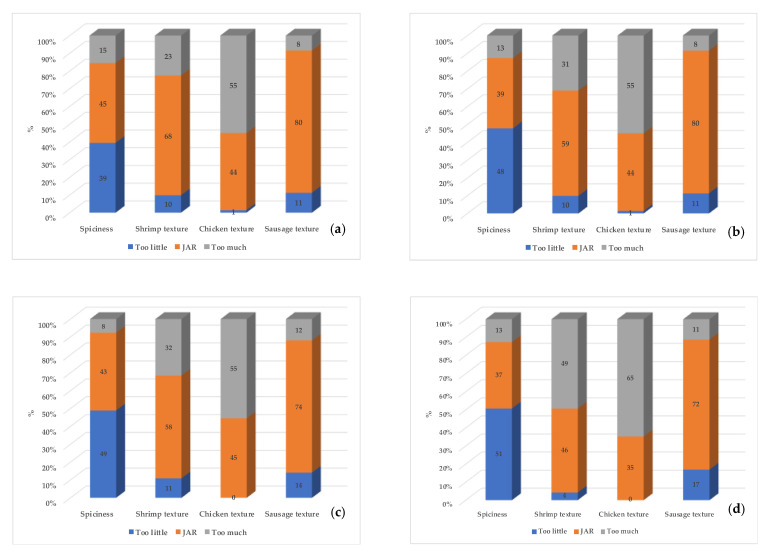
Percentage of responses for the indicated anchor of the JAR scale in the sensory evaluation of the intensity of spiciness and texture of the three meat components of the jambalaya meals (MAPS-processed and control) over a 12-week storage period (*n* = 71). (**a**) Control after 2 weeks of storage; (**b**) Control after 12 weeks of storage; (**c**) MAPS-jambalaya after 2 weeks of storage; (**d**) MAPS-jambalaya after 12 weeks of storage.

To evaluate the impact of the processing methods and storage time on the spiciness and texture perception of the meat components of the meals, JAR questions were used. The main results are presented in Figure 3.

JAR questions are useful as they can provide focused direction to new product development. With penalty analysis, it is possible to determine which elements most impact the overall liking of a product.

The spiciness intensity in most of the samples was considered not spicy. The texture of the shrimp and sausage were mostly rated “just-about-right” on the JAR scale for both the control and the MAPS-processed jambalaya. In addition, when the first evaluation time point (2 weeks) was compared to the last one (12 weeks), the ratings were similar. Chicken texture was the one attribute most penalized by the participants of the study as they considered it to be too chewy/overcooked. This was observed for both the control and the MAPS-processed jambalaya in both time points, after 2 and 12 weeks of storage, respectively.

To complement the potential improvements that could be made to the jambalaya meals, on the last evaluation time point the participants (*n* = 50) answered a series of JAR questions.

Most of the aspects of the meals were rated close to two on the three-point scale, which corresponds to the JAR point. The other two values on the scale were 1 (=less than I would like) and 3 (=more than I would like).

The portion size was scored with an average value of 1.68, the quantity of sauce was rated with 1.96, the quantity and size of vegetables were scored with 1.56 and 1.82, respectively. The quantity of shrimp, chicken and sausage with 1.74, 2.12 and 1.72. The saltiness was rated with a value of 2.04, also very close to the JAR point.

The only question that pointed out a potential improvement was the question related to removing the tails of the shrimps (score = 2.84). Most of the participants favored the removal of the tails.

Overall, JAR questions contributed on having a better understanding of Hypothesis 1.

### 3.2. Semi-Trained Panel Evaluation

The other evaluation was done by a semi-trained panel. This panel used RATA questions as the sensory evaluation technique. This component of the study also supported Hypothesis 1 of the study.

For the analysis, the effect of the treatment (MAPS-processed vs. control), the effect of the storage time, and their respective interaction on the intensity of different sensory attributes were evaluated. The interaction was not significant for any the sensory modalities; thus, the simple effects were analyzed.

As shown in Table 8, only the storage significantly impacted the intensity of different sensory attribute scores as evaluated by the panelists.

The aroma related attributes, for example oxidized and brothy-chicken significantly increased as storage time increased. In the case of oxidized aroma, the values ranges were less than 1; therefore, they are considered to be more in the low range. The brothy-chicken aroma increased mainly after 8 weeks of storage. Barnett et al. (2019) [23] reported increases in the intensities of some aroma and flavor attributes of microwave-processed Cajun chicken pasta meals as the storage time increased. The concentration of aroma and flavor may have resulted from potential water migration from the package [23,37].

For the appearance related attributes, oily appearance intensity significantly increased due to the effect of the storage time, meanwhile the shriveled/overcooked appearance in chicken decreased. This result could be explained by the fact that this specific attribute could be a difficult one to evaluate, therefore the panelists gave initially medium to high score and then after 8 weeks of storage a score between low and medium. The opposite trend could have been expected, that as an effect of the potential water migration in the tray [23,37] the chicken experienced some level of dehydration and therefore was perceived by the panelists as more shriveled-overcooked.

Another appearance attribute that was significantly different was the shriveled-overcooked appearance of the shrimp. In this case the interaction processing method × storage time was significant. At 2 weeks of storage the MAPS-processed jambalaya (2.20) was rated significantly higher (*p* = 0001) as the control (0.80). This result could be explained by the fact that the MAPS-processed meal goes through an extra thermal processing step. However, at 8 and 12 weeks of storage there were not significant differences when comparing the MAPS-processed jambalaya to the control. The intensities of shriveled-overcooked appearance of the shrimp at 8 and 12 weeks of storage ranged between 2.00 and 1.50.

It is also important to consider that these two texture-related appearance attributes, shriveled/overcooked appearance in chicken and shriveled-overcooked appearance of the shrimp could have more impact if they significant difference in the texture sensory modality and that was not the case.

The final type of sensory attribute that was impacted by storage time was the texture of the sausage, specifically its chewiness. This attribute significantly increased as storage time increased primarily when Week 2 and Week 8 of storage were compared.

Overall, based on the RATA results few sensory attributes were affected by the main factors evaluated in the study.

## 4. Conclusions

Our findings showed that the acceptance/liking of different sensory characteristics of MAPS-processed jambalaya did not change significantly during storage at 2 °C as compared to a control (cooked and frozen jambalaya stored at −31 °C) over a 12-week storage period. Using a HUT to evaluate consumer acceptance of jambalaya and including partner participation, is a promising way of testing acceptance in a more realistic context. An on-line auction with a HUT sensory testing of jambalaya meals showed that consumers are willing to pay a price that is comparable to commercially available jambalaya meals. Both were ecologically valid measures of consumer acceptance and positively impacted the degree of acceptance of the RTE meals.

This study has utility and presents an innovative approach as compared to previously reported HUTs. The inclusion of a partner in the evaluation of a ready-to-eat meal processed with a novel technology, such as microwave-assisted pasteurization has not been previously reported. In addition, the evaluation was conducted during an extended period of time of 12 weeks. Most reported HUTs are conducted within a week and the exposure to the product being tested occurs one time or consecutive times within a short period of time (e.g., one week). In addition, the study incorporated the evaluation of a complimentary evaluation with a semi-trained panel that used a rapid method, rate-all-that-apply. These elements were combined with an online auction to determine consumers’ willingness to pay for the meals.

As for the HUT results, it should be noted that this study had an observational/exploratory approach, focused on a specific RTE meal. Because of the relatively restricted number of consumers that joined the study, future research should evaluate the effect of the inclusion of partner as an intentional/designated treatment and target a larger number of participants. A HUT and an SST (laboratory test or central location test) could be conducted simultaneously at a defined storage period and the results of both tests compared.

In addition, the inclusion of an analytical technique, like gas chromatography-mass spectrometry and texture profile analysis (TPA), could be considered for future studies to describe specific chemical and physical changes in the meals. However, multicomponent meals are a complex/challenging food matrix to work with that is why sensory evaluation seems like a more effective methodology to characterize them during storage instead of analytical techniques.

## Figures and Tables

**Table 1:** Jambalaya formulation and ingredient specifications.

Ingredient	Percentage (%)	Source/Brand
Vegetables		
Crushed tomatoes	34.12	Signature Select
Pre-chopped yellow onion	8.77	Safeway brand
Pre chopped celery ribs	4.09	Safeway brand
Pre chopped *pasilla* pepper	4.02	Safeway brand
Ready-to-use minced garlic	0.66	Spice world
Meat		
Chicken breast	13.32	Safeway brand
Raw, small, shell and tail-on shrimp	9.97	Waterfront Bistro
Andouille pork smoked sausages	9.03	Johnsonville
Spices		
Worcestershire sauce	0.64	Lea and Perrin
Old Bay seasoning	0.11	McCormick
Cajun seasoning	0.10	IGA brand
Salt	0.05	Morton
Coarse ground black pepper	0.03	McCormick
Other		
Chicken broth	15.09	Swanson
Total	100.00	

**Table 2:** Preparation and processing schedule of the three batches of jambalaya used in the study.

Steps in the Process	Day 1	Day 2	Day 3	Day 4
Cooking/assembling	B1 ^1^	B2 ^2^	B3 ^3^	
MAPS processing		B1	B2	B3
Freezing of the controls		B1	B2	B3

^1^ B1: Batch 1; ^2^ B2: Batch 2; ^3^ B3: Batch 3.

**Table 3:** Pathogens and spoilage-related microbial analyses conducted on the MAPS-processed jambalaya and the control during 12 weeks of storage at 2 °C and −31 °C, respectively.

Storage Time (Weeks)	Treatment	Microorganism Tested						
		*B. cereus* (CFU/g)	*Salmonella* (in 25 g)	*L. monocytogenes* (in 25 g)	*E. coli* O157:H7 (in 25 g)	Aerobic Count (CFU/g)	Yeast and Molds (CFU/g)	Total Coliforms (CFU/g)
2	MAPS ^1^	<10	Negative	Negative	Negative	40	<10	<10
	Control	<10	Negative	Negative	Negative	10	<10	<10
8	MAPS	<10	Negative	Negative	Negative	10	<10	10
	Control	<10	Negative	Negative	Negative	21	<10	<10
12	MAPS	<10	Negative	Negative	Negative	<10	<10	<10
	Control	<10	Negative	Negative	Negative	86	<10	<10

^1^ Microwave-thermally processed jambalaya is represented as MAPS and cooked frozen jambalaya is represented as Control.

**Table 4:** Consumer liking responses and bids’ values for jambalaya MAPS-processed meals and a control (cooked and frozen meals) as evaluated by 50 and by 71 participants.

Processing Method		Appearance	Aroma	Flavor	Texture Shrimp	Texture Chicken	Texture Sausage	Overall liking	Bids ($)
*n* = 50	MAPS-processed	5.66 a	5.79 a	6.00 a	5.13 a	4.95 a	5.68 a	5.61 a	3.59 a
	Control	5.71 a	5.92 a	5.99 b	5.35 a	4.94 a	5.70 a	5.67 a	3.48 a
	*p*-value	0.78	0.40	0.94	0.32	0.98	0.92	0.70	0.48
*n* = 71	MAPS-processed	5.63 a	5.68 a	5.88 a	5.16 a	4.93 a	5.63 a	5.34 a	-
	Control	5.70 a	5.85 a	5.89 a	5.27 a	4.98 a	5.77 a	5.65 a	-
	*p*-value	0.64	0.23	0.96	0.54	0.78	0.34	0.45	-

Different letters within a column (a, b) indicate that the tested parameter mean value was different among processing methods *p* < 0.05 as determined by using Tukey’s HSD. Mean values are collapsed over participants and storage time. Results range between 1 and 7 due to the use of a 7-point hedonic scale.

**Table 5:** Consumer liking responses and bid values for jambalaya meals as evaluated by 50 and by 71 participants, over a 12-week storage period.

Storage Time (Weeks)		Appearance	Aroma	Flavor	Texture Shrimp	Texture Chicken	Texture Sausage	Overall Liking	Bids ($)
*n* = 50	2	5.77 a	5.85 a	6.29 a	5.30 a	5.14 a	5.80 a	5.72 a	3.54 a
	8	5.69 a	5.80 a	5.80 b	5.43 a	5.00 a	5.71 a	5.59 a	3.42 a
	12	5.79 a	5.91 a	5.89 b	5.00 a	4.69 a	5.56 a	5.61 a	3.65 a
	*p*-value	0.26	0.53	<0.0001	0.03	0.02	0.20	0.46	0.50
*n* = 71	2	5.55 a	5.74 a	6.09 a	5.16 a	5.12 a	5.78 a	5.68 a	-
	8	5.63 a	5.72 a	5.75 b	5.41 a	5.01 a	5.73 a	5.55 a	-
	12	5.82 a	5.83 a	5.81 ab	5.09 a	4.75 a	5.59 a	5.54 a	-
	*p*-value	0.004	0.45	0.001	0.06	0.03	0.22	0.26	-

Different letters within a column (a, b) indicate the mean value of the tested parameter was different across storage times at *p* < 0.05 as determined using Tukey’s HSD. Mean values are collapsed over participants and processing method. Results range between 1 and 7 due to the use of a 7-point hedonic scale.

**Table 6:** Consumer liking responses and bid values for jambalaya meals as evaluated by participants who had or did not have a partner (*n* = 50).

Partner	Appearance	Aroma	Flavor	Texture Shrimp	Texture Chicken	Texture Sausage	Overall Liking	Bids ($)
Yes	5.88 a ^1^	5.98 a	6.04 a	5.40 a	5.07 a	5.83 a	5.69 a	3.59 a
No	5.54 b	5.76 a	5.96 a	5.13 a	4.85 a	5.59 a	5.60 a	3.50 a
*p*-value	0.04	0.18	0.63	0.24	0.35	0.20	0.62	0.57

^1^ Different letters within a column (a,b) indicate that the tested parameter mean value was different among those participants with a partner vs. those without at *p* < 0.05 as determined using Tukey’s HSD. Data are collapsed over processing method and storage time. Results range between 1 and 7 due to the use of a 7-point hedonic scale.

**Table 7:** Categories, frequency of mention and some comment examples from the participants of the study (*n* = 50).

Categories	Frequency of Mention (%)	Comment Examples
Enjoyed experience with partner	26	“I enjoyed sharing this with my partner, who was equally eager to partake due to being unable to partake in the in-lab studies” “Nice to do the tasting a home on my own time and get feedback from my wife” “Sharing the samples with my partner is enjoyable, because after we record our ratings individually, we compare and discuss the two samples” “I enjoyed having this experience with my partner as we could discuss what we liked and didn’t like”
HUT vs. in-lab evaluation	42	“Way more convenient than in-lab and it’s the same setting in which I’d be eating the convenience meal if I purchased one” “I liked being able to utilize my samples as dinner on each of the nights. I felt that I could give a better evaluation because I had more of a sample to taste” “I really appreciate the research these students are involved in and the fact they incorporate real people and samples rather than all lab studies” “I like bringing the samples home to taste because it is more realistic and relevant to how I would actually eat the food”
Time flexibility	14	“This in-home study offered me more flexibility with the time period. I didn’t feel pressured to get out of my chair and let the next person in line have a seat” “I spend more time evaluating at home versus in the lab where it is hurried to leave” “I liked being able to take my time and enjoy each sample without feeling rushed” “I enjoyed having more flexibility in the amount of time I could take the survey”
Fun/positive experience	42	“My husband and I had fun with your in-home study, thank you” “It was a pleasant in-home study, and the quality was good” “I enjoyed being able to do this at home” “This was fun to participate in and it was easy”
Liking of the meals	16	“I very much enjoyed the samples and will be sad to not have them anymore” “I enjoyed the food and getting cash for participating” “…the jambalaya was something I looked forward to, I didn’t get tired of it” “I could have eaten both samples myself”
Willingness to pay for the meals	8	“We both liked the meals and would definitely purchase if available at the store” “I hope I will see the samples in the store” “I could better consider if the sample would be something I would purchase” “We would be willing to spend a little more on these since they have a large variety in them…”

**Table 8:** Mean intensity values of multiple sensory attributes of jambalaya meals as evaluated by a semi-trained panel (*n* = 10) over a 12-week storage period with RATA.

Storage Time (Weeks)	Oxidized Aroma	Brothy-Chicken Aroma	Oily Appearance	Shriveled/Overcooked Chicken Appearance	Chewy Sausage
2	0.05 a	2.15 a	0.25 a	2.45 a	1.55 a
8	0.10 b	2.85 b	1.50 b	1.50 b	2.40 b
12	0.68 ba	2.60 ab	1.72 b	1.15 b	2.01 ab
*p*-value	<0.0001	0.002	<0.0001	<0.0001	0.001

Different letters within a column (a,b) indicate that attribute intensities were different among storage times at *p* < 0.05 as determined using Tukey’s HSD. These results range between 0 and 3 due to the use of a four-point scale of 0, 1, 2, 3 (absent, low, medium, high). Mean values are collapsed over processing method, replicate and panelists.

## Data Availability

The data that support the findings of this study are available from the corresponding author upon reasonable request. The data are not publicly available due to privacy issues.
